# Identification of senescence-related subtypes, establishment of a prognosis model, and characterization of a tumor microenvironment infiltration in breast cancer

**DOI:** 10.3389/fimmu.2022.921182

**Published:** 2022-08-22

**Authors:** Yanling Zhou, Liang Xiao, Guo Long, Jing Cao, Shuang Liu, Yongguang Tao, Ledu Zhou, Jianing Tang

**Affiliations:** ^1^ Department of Oncology, Institute of Medical Sciences, National Clinical Research Center for Geriatric Disorders, Xiangya Hospital, Central South University, Changsha, China; ^2^ Department of Liver Surgery, Xiangya Hospital, Central South University, Changsha, China; ^3^ National Clinical Research Center for Geriatric Disorders, Xiangya Hospital, Central South University, Changsha, China; ^4^ Department of Breast, Xiangya Hospital, Central South University, Changsha, China; ^5^ Key Laboratory of Carcinogenesis and Cancer Invasion, Ministry of Education, Department of Pathology, Xiangya Hospital, Central South University, Changsha, China; ^6^ Key Laboratory of Carcinogenesis of the Ministry of Health, Cancer Research Institute, School of Basic Medicine, Central South University, Changsha, China; ^7^ Department of Thoracic Surgery, Hunan Key Laboratory of Tumor Models and Individualized Medicine, Second Xiangya Hospital, Central South University, Changsha, China

**Keywords:** breast cancer, senescence, tumor microenvironment, prognosis model, immune infiltration

## Abstract

Breast cancer is a malignancy with the highest incidence and mortality in women worldwide. Senescence is a model of arrest in the cell cycle, which plays an important role in tumor progression, while the prognostic value of cellular senescence-related genes (SRGs) in evaluating immune infiltration and clinical outcomes of breast cancer needs further investigation. In the present study, we identified two distinct molecular subtypes according to the expression profiles of 278 SRGs. We further explored the dysregulated pathways between the two subtypes and constructed a microenvironmental landscape of breast cancer. Subsequently, we established a senescence-related scoring signature based on the expression of four SRGs in the training set (GSE21653) and validated its accuracy in two validation sets (GSE20685 and GSE25055). In the training set, patients in the high-risk group had a worse prognosis than patients in the low-risk group. Multivariate Cox regression analysis showed that risk score was an independent prognostic indicator. Receiver operating characteristic curve (ROC) analysis proved the predictive accuracy of the signature. The prognostic value of this signature was further confirmed in the validation sets. We also observed that a lower risk score was associated with a higher pathological response rate in patients with neoadjuvant chemotherapy. We next performed functional experiments to validate the results above. Our study demonstrated that these cellular senescence patterns effectively grouped patients at low or high risk of disease recurrence and revealed their potential roles in the tumor–immune–stromal microenvironment. These findings enhanced our understanding of the tumor immune microenvironment and provided new insights for improving the prognosis of breast cancer patients.

## Introduction

Breast cancer is one of the most common cancers worldwide, with 2.2 million cases (11.7% of all cancer cases) in 2020 (1). Breast cancer is a heterogeneous disease with multiple molecular features (2). Based on the expression of estrogen receptor (ER), progesterone receptor (PR), and human epidermal growth factor receptor 2(HER2), there are at least four molecular subtypes of breast cancer: luminal, basal, human epidermal growth factor receptor 2 (HER2)‐enriched, and normal‐like (3). With the development of surgery, chemotherapy, endocrine therapy, and targeted therapy, the prognosis of breast cancer patients has been improved (1). However, due to the heterogeneity of patients, the benefits of these treatments are limited. Therefore, it is imperative to further understand the molecular mechanisms underlying breast cancer progression and to explore more effective strategies.

Senescence, a state of permanent cell cycle arrest in response to mitogens and oncogenic transformation, is vital to aging research and tumor progression (4–6). The occurrence of senescence involves the engagement of DNA damage response (DDR), the accumulation of cyclin-dependent kinase inhibitors (CDKi), the alteration of metabolic rates, and the stress on the endoplasmic reticulum (ER) (4, 7). Meanwhile, senescent cells show structural changes, including the enlargement of the cell body, the different compositions of the plasma membrane (PM), the accumulation of lysosomes and mitochondria, and changes within the nucleus (8). Senescent cells can secrete chemokines, growth factors, inflammatory cytokines, and matrix metalloproteinases, which is called senescence-associated secretory phenotype (SASP) (9, 10). Moreover, overexpression of p16^INK4A^, p53, p21^CIP1^, and hypophosphorylated RB is used as senescence biomarkers (6, 11). Considering its therapeutic potential, cellular senescence has emerged as a potent tumor suppression mechanism that restrains proliferation in cells at risk for malignant transformation. Recent studies also revealed the dual role of senescence in malignant transformations. Senescence, glycolysis, and autophagy are a continuum of the same biological spectrum, all generating a “fertile” tumor microenvironment that sustains breast cancer tumor growth (12). In the early stage of the lesion, higher levels of p53 and p16 and lower levels of Ki-67 are related to the upregulation of SA-β-ga, a senescence biomarker, which suggested the protection effects of senescence in the early stage of tumorigenesis (13). Therefore, compounds that stimulate the growth inhibition effects of senescence while limiting its detrimental effects are believed to have great clinical potential (14). SASP factors can promote angiogenesis, proliferation, and epithelial–mesenchymal transition of tumors through paracrine or autocrine mechanisms. However, SASP can also have antitumor effects by inducing the senescence of surrounding tumor cells (15). Although the main role of senescence is thought to be related to tumor suppression, detailed studies are needed to characterize the exact role of senescence in cancer.

The tumor microenvironment (TME) consists of innate immune cells, including macrophages, neutrophils, dendritic cells, innate lymphoid cells, myeloid-derived suppressor cells (MDSCs), natural killer cells, and adaptive immune cells including T cells and B cells (16). The TME influences tumor initiation and invasion and plays a vital role in therapeutic efficacy (17). Growing evidence also shows the interaction between senescence and TME. Senescent cells are proven to be cleared by the humoral immune system and various immune cells, including natural killer cells (NKs), macrophages, and T cells (18). Meanwhile, DNA damage responses caused by Treg cells and tumor cells result in cell cycle arrest and senescence (19). Moreover, senescent T cells possess suppressive activity, boosting the immune suppression in the TME (20). SASP-driven secondary senescence induced by other senescent cells within the TME can promote the development of senescence in immune cells (18). Zhao et al. found that high levels of p16^INK4a^ in T cells indicate the worst prognosis, suggesting that the correlation between the TME and senescence benefits the prognostic indicator (21). Therefore, a detailed understanding of senescence may provide profound insights into the tumorigenesis of breast cancer and improve the response to immunotherapy.

To systematically assess the correlations between senescence and the prognosis of breast cancer, we evaluated the profiles of senescence-related genes (SRGs) and obtained a comprehensive overview of the immune landscape. Firstly, 252 breast cancer patients from GSE21653 were divided into two subtypes according to the expression profiles of 279 SRGs. We then established a scoring system to predict relapse-free survival (RFS) and characterized the immune landscape of breast cancer, which may be beneficial for personalized therapeutic strategies.

## Methods

### Data processing

Gene expression and the related prognostic and clinical information of GSE21653, GSE20685, and GSE25055 were obtained from the Gene Expression Omnibus (GEO). GSE21653 contained 266 early breast cancer patients who underwent initial surgery, and the gene expression data of 266 breast cancers were quantified by using whole-genome DNA microarrays (HG-U133 plus 2.0, Affymetrix Santa Clara, USA). GSE20685 contained 327 breast cancer samples; 268 patients underwent adjuvant chemotherapy and 91 patients had a relapse. GSE25055 contained 310 HER2-negative breast cancer cases treated with taxane–anthracycline chemotherapy preoperatively and endocrine therapy if ER-positive. The patients with complete survival information were included in our analysis. Raw microarray cell intensity files were preprocessed using the Robust Multichip Average package in R. The RNA expression data were scaled with a standard deviation of 1. A manually curated gene list including 278 cellular senescence-related genes was extracted from the CellAge database (https://genomics.senescence.info/cells/, **Supplementary Table 1**).

### Consensus clustering analysis of senescence-related genes

We performed a consensus unsupervised clustering analysis of senescence-related genes by the R package “ConsensusClusterPlus” and classified patients into distinct molecular subtypes. This clustering was performed according to the following criteria: firstly, the cumulative distribution function (CDF) curve increased gradually and smoothly. Secondly, each group had a suitable sample size. Lastly, the intragroup correlation grew in number through clustering, while the correlation of intergroup declined. Principal component analysis (PCA) was conducted using the prcomp command of the R statistical software.

### Enrichment analysis

Gene Ontology (GO) and Kyoto Encyclopedia of Genes and Genomes (KEGG) analysis for cellular senescence-related genes were performed using the R package “clusterProfiler.” To calculate the relevance of senescence-related genes and the activity of oncogenic pathway activity in breast cancer, the well-defined 50 cancer hallmark-related pathways gene sets were collected from the Molecular Signature Database of Gene Set Enrichment Analysis (hallmark gene sets, http://www.gsea-msigdb.org/gsea/msigdb). Gene set variation analysis (GSVA) was also performed with the R package “GSVA” to calculate the enrichment score of each pathway. The gene sets of “h.all.v7.2.symbols” downloaded in MSigDB and the known gene sets constructed by Mariathasan et al. were used for GSVA enrichment analysis.

### Construction of the senescence‐related prognostic signature

To establish a predictive model for cancer prognosis, univariate Cox regression analysis was first performed to identify prognostic genes. *p <*0.05 was considered significant. Then, a popular method for variable selection—the least absolute shrinkage and selection operator (LASSO) method for variable selection in a Cox regression model—was used to select the most useful prognostic genes with the R package “glmnet.” The senescence‐related signature for patients with breast cancer was built by considering the genes’ expression and correlation-estimated Cox regression coefficients: risk score = Σ(expression of gene * coefficient of gene).

### Relationship between senescence‐related signature and prognosis of breast cancer

To examine the prognostic value of the senescence-related signature, we compared the relationships between the senescence-related signature and prognosis. The differences in RFS were assessed using Kaplan–Meier curves generated by the “survival” and “survminer” R packages. Furthermore, we used GSE25055 to determine whether the scores were associated with treatment outcomes. GSE25055 is a neoadjuvant study of 310 HER2-negative breast cancer cases treated with taxane–anthracycline chemotherapy preoperatively and endocrine therapy if ER-positive. The risk scores were calculated in different pathological response groups.

### The immune phenotype of breast cancer

To understand the immune status of breast cancer patients, single-sample gene set enrichment analysis (ssGSEA) was used to assess the abundance of immune cells of each sample in GSE21653 by a gene set of 28 immune cell types. Stromal and immune cells were assessed through ESTIMATE (Estimation of Stromal and Immune cells in Malignant Tumor tissues using Expression Data).

### RNA inference

Small interfering RNAs targeting CPEB1 (siG000064506A-1-5), NOTCH3 (siG09820100759-1-5), NUAK1 (siG000009891A-1-5), and PDPK1 (siG000005170A-1-5) were obtained from Ruibo Biotechnology Co., Ltd. (Guangzhou, China). Lipofectamine 2000 (Invitrogen, Carlsbad, CA, USA) was used for siRNA transfection according to the manufacturer’s instructions. In brief, MDA-MB-231 cells were seeded to be 70%–90% confluent at transfection. Lipofectamine 2000 reagent (5 µl) and 5 µl of siRNA (10 μM) were mixed in 250 µl of Opti-MEM medium. The mixture was then incubated at room temperature for 10 min and then added dropwise into a culture dish containing 1 ml of the medium. Transfected cells were cultured under normal culture conditions (5% CO_2_, 37°C) for 24 h. After that, the cells were digested and resuspended for further experiments.

### Colony formation and migration analysis

For the colony formation assay, the cells were treated with the indicated siRNAs for 24 h, digested, and seeded into six-well plates at a density of 1,000 cells per well. After 14 days of incubation, the cells were fixed with 4% paraformaldehyde and visualized by 0.5% crystal violet staining. Cell migration capacity was assessed using 8-μm pore polycarbonate membrane Transwell plates (Corning, USA). Briefly, 5 × 10^5^ cells were suspended without serum and were seeded into the upper chambers precoated with Matrigel (BD BioCoat, USA). The bottom chambers were filled with 600 μl of complete medium. After 24 h, the cells on the bottom side of the pore membrane were fixed and stained with crystal violet.

### Immunohistochemistry staining for breast cancer samples

Tissue microarray (TMA) was collaborated with Alenabio Technology Co., Ltd. (Xian, China). The tissue microarray contained 138 breast cancer specimens. Briefly, paraffin sections were first deparaffinized, antigen retrieval was performed in citrate buffer (pH 6.0), and endogenous peroxidase activity was blocked in 0.3% H_2_O_2_. The slides were continuously incubated with the indicated primary and secondary antibodies until visualization with peroxidase and 3,3′-diaminobenzidine tetrahydrochloride. The expression of CPEB1, NOTH3, NUAK1, and PDPK1 in the breast cancer tissues from the tissue microarray was blindly quantified by two pathologists based on histochemical score (H-score) as previously described (22). The primary antibodies were CPEB1 (Proteintech, Wuhan, China 13274-1-AP), NOTCH3 (Proteintech, Wuhan, China 55114-1-AP), NUAK1 (Proteintech, Wuhan, China 22723-1-AP), and PDPK1 (Proteintech, Wuhan, China 17086-1-AP).

### Statistical analysis

Kaplan–Meier curve analysis with a two-tailed log-rank test was performed to evaluate the prognostic significance. To clarify whether the senescence-related score is an independent prognostic factor, the R package “survival” was used for multivariate Cox regression analysis. The chi-square test was introduced to calculate the between-group differences. R software (version 3.5.1) (https://www.r-project.org) was used for the data processing and analysis. A significant difference in all statistical methods of this study was considered if the *p*-value was less than 0.05.

## Results

### Identification of senescence subtypes in breast cancer

To fully comprehend the profile of senescence-related genes in breast cancer tumorigenesis, 252 patients from GSE21653 were selected for further analysis in our research. Detailed information of the 252 breast cancer patients is presented in **Supplementary Table 2**.

**Figure 1:**
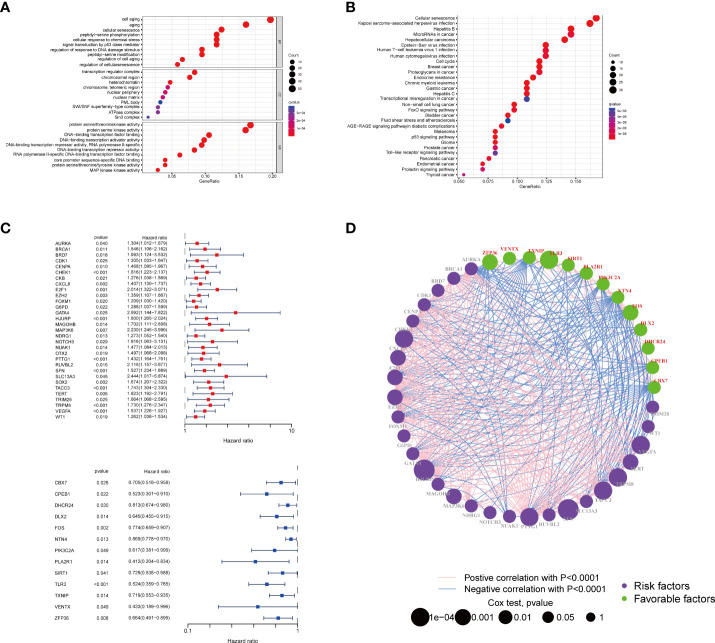
Enrichment analysis of the differently expressed genes and the PPI of the two groups. The bubble plot of the biological process **(A)** and pathways **(B)** enriched between the two subtypes. **(C)** The forest plot of the survival analysis of the senescence-related genes. **(D)** Interactions among senescence-related genes in BRCA. The line connecting the senescence-related genes represents their interaction, with the line thickness indicating the strength of the association between senescence-related genes. Green and pink represent negative and positive correlations, respectively. PPI, protein–protein interaction; BRCA, breast cancer.

We first conducted a functional enrichment analysis to explore the potential biological functions of senescence-related genes. As expected, these senescence-related genes were significantly enriched in biological processes like cell aging and senescence (**Figure 1A**). In addition, KEGG analysis indicated that senescence-related genes were significantly enriched in cancer-related pathways, including senescence, cell cycle, and endocrine resistance (**Figure 1B**). Then, univariate Cox regression was performed to assess the prognostic values of the 278 senescence-related genes in patients with breast cancer. Genes were divided into high and low expression according to the median expression level. We also identified 44 genes that were significantly associated with the patients’ survival. Among them, 13 senescence-related genes were associated with poor survival, and the other 31 genes were associated with better prognosis (**Figure 1C**). The vast landscape of senescence-related gene interactions and their prognostic value in patients with breast cancer patients were demonstrated in a network (**Figure 1D**).

**Figure 2:**
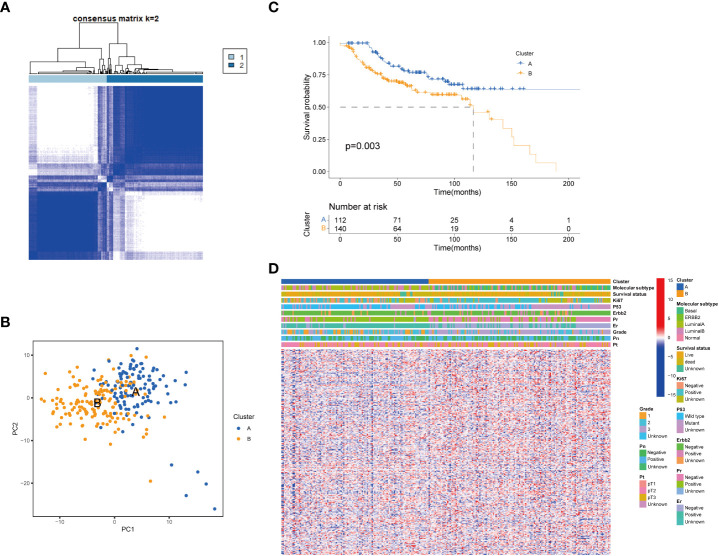
Senescence-related genes and the clinicopathological and biological characteristics of the two distinct subtypes of samples divided by consistent clustering. **(A)** Consensus matrix heatmap defining two clusters (*k* = 2) and their correlation area. **(B)** PCA analysis showing a remarkable difference in transcriptomes between the two subtypes. **(C)** Univariate analysis showing 278 senescence-related genes related to the RFS time. **(D)** Differences in clinicopathologic features and expression levels of senescence-related genes between the two distinct subtypes. PCA, principal components analysis; RFS, recurrence-free-survival.

To further clarify the traits of senescence-related genes in breast cancer, we used a consensus clustering algorithm to categorize the 252 breast cancer patients based on the expression profiles of the 278 senescence-related genes. We sorted the entire cohort into two subtypes: clusters A (*n* = 112) and B (*n* = 140), which meant *k* = 2 proved to be a preferable choice (**Figure 2A**). The results of the PCA analysis indicated distributed discrete directions of senescence-related genes between two clusters (**Figure 2B**). Patients in cluster A exhibited a longer RFS compared to those in cluster B according to the analysis of Kaplan–Meier curves (log-rank test, *p* = 0.003; **Figure 2C**). Furthermore, we observed that patients in the two clusters exhibited significantly different clinicopathological features, including molecular subtypes, tumor grade, T stage, N stage, P53 mutation, and the expression of Ki-67, HER2, PR, and ER (**Figure 2D**; **Table 1**). As shown in **Table 1**, the median follow‐up was 66.1 months in cluster A and 43.8 months in cluster B. In cluster A, patients are more likely to possess lower tumor grade, positive ER status, positive PR status, low frequency of p53 mutation, and negative Ki-67 status than those in cluster B. When we stratified the patients by clinicopathologic factors, this senescence-related subtype could not independently predict the prognosis. As shown in **Supplementary Figure 1**, cluster B tumors often have a poor prognosis in patients with small tumor size (≤2 cm, *p* = 0.044, HR = 1.8), advanced tumor grade (grade III, *p* = 0.0029, HR = 2.3), or triple-negative tumor (*p* = 0.0075, HR = 2.1) when controlling for the remaining clinicopathologic factors.

**Table 1:** Clinicopathologic characteristics of breast cancer patients according to the senescence pattern.

Variables	GSE21653		*p*-value
	Cluster A (%)	Cluster B (%)	
Age at diagnosis (years)			0.657
≤50	37 (33.0)	50 (35.7)	
>50	75 (67.0)	90 (64.3)	
Tumor size			0.405
T1	29	28	
T2	55	66	
T3	26	40	
Unknown	2	6	
Lymph node status			0.488
Negative	49 (44.1)	67 (48.6)	
Positive	62 (55.9)	71 (51.4)	
Grade			<0.001
I	35 (31.5)	8 (5.8)	
II	54 (48.6)	30 (21.9)	
III	22 (19.8)	99 (72.3)	
ER status			<0.001
Negative	12 (10.8)	98 (70.5)	
Positive	99 (89.2)	41 (29.5)	
PR status			<0.001
Negative	21 (18.9)	103 (74.1)	
Positive	90 (81.1)	36 (25.9)	
HER2 status			<0.001
Negative	98 (87.5)	109 (77.9)	
Positive	3 (2.7)	23 (16.4)	
Unknown	11 (9.8)	8 (5.7)	
P53 status			<0.001
Wild type	76 (67.9)	49 (35.0)	
Mutant	18 (16.1)	50 (35.7)	
Unknown	18 (16.1)	41 (29.3)	
Ki-67 status			<0.001
Negative	44 (39.3)	14 (10.0)	
Positive	46 (41.1)	96 (68.6)	
Unknown	22 (19.6)	30 (21.4)	
Molecular subtype			<0.001
Basal	1 (0.9)	74 (52.9)	
HER2	1 (0.9)	21 (15.0)	
Luminal A	72 (64.3)	13 (9.3)	
Luminal B	25 (22.3)	19 (13.6)	
Normal	13 (11.6)	13 (9.3)	
Vital status			0.063
Alive	82 (73.2)	87 (62.1)	
Dead	30 (26.8)	53 (37.9)	
Median follow-up (months)	66.1	43.8	

ER, estrogen receptor; PR, progesterone receptor; HER2, human epidermal growth factor receptor 2; P53, tumor protein P53; Ki-67, proliferation marker protein Ki-67.

### Characteristics of TME in distinct subtypes

**Figure 3:**
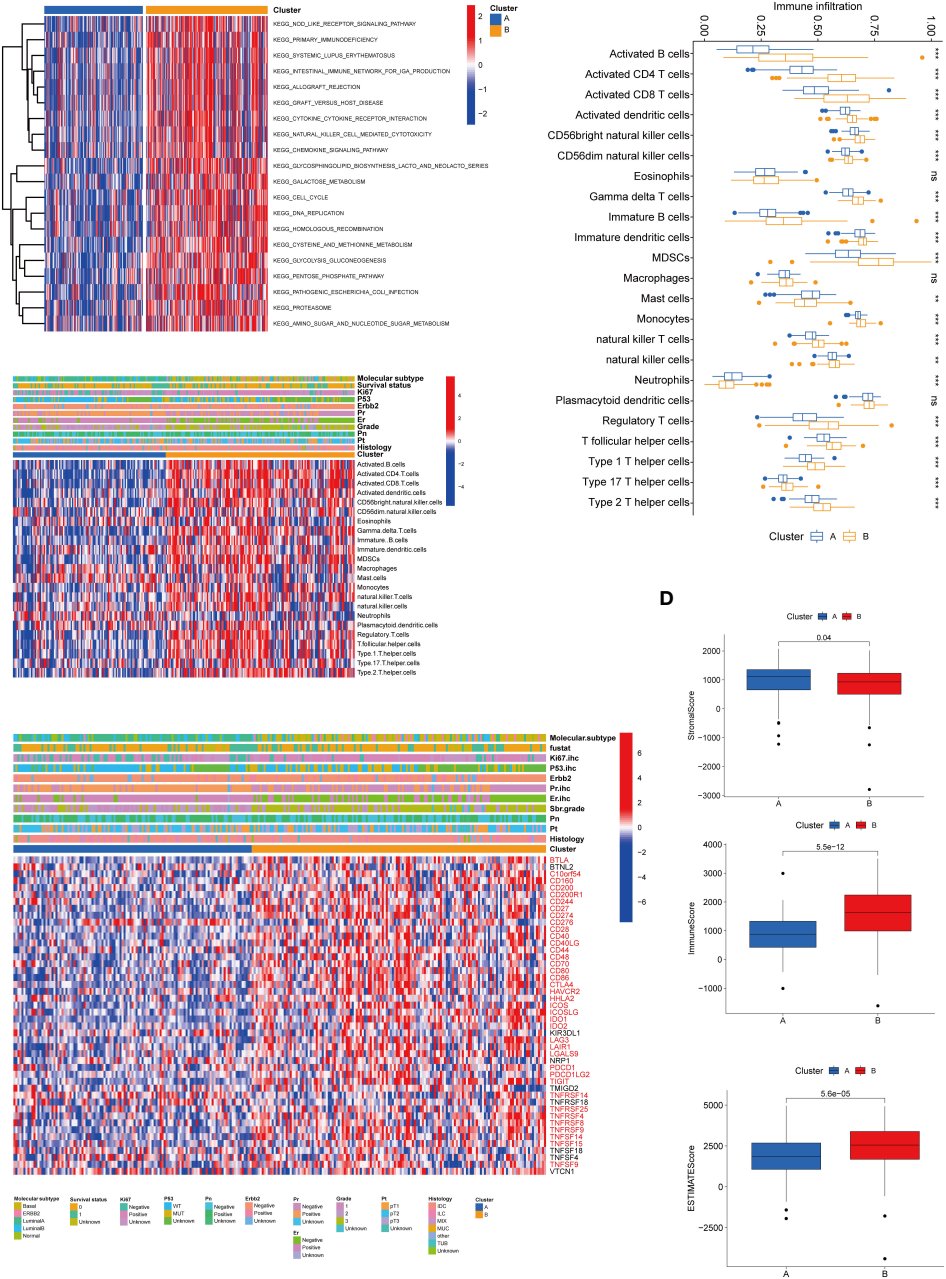
Correlations of tumor immune cell microenvironments and two BRCA subtypes. **(A)** GSVA of biological pathways between the two distinct subtypes, in which red and blue represent activated and inhibited pathways, respectively. **(B)** Heatmap of the tumor-infiltrating cell in the two BRCA subtypes. **(C)** The abundance of the 23 infiltrating immune cell types in the two BRCA subtypes. **(D)** Correlations between the two subtypes and TME score. **(E)** Heatmap of immune checkpoints between the two distinct subtypes, in which red represents differently expressed checkpoints. BRCA, breast cancer; GSVA, gene set variation analysis; TME, tumor microenvironment. **P value < 0.01; ***P value < 0.001.

To further clarify the dysregulated pathways between the two clusters, we next conducted a GSVA enrichment analysis. We found that cluster B was significantly enriched in numerous immune pathways, including NOD-like receptor signaling pathway, primary immunodeficiency, and intestinal immune network for IgA production and graft versus host disease, suggesting that senescence may play a role in the immune regulation of the TME (**Figure 3A**). We then performed a GSEA analysis and found that biological functions related to cellular senescence and aging were significantly enriched in cluster A (**Supplementary Figure 2A**). To comprehensively explore the associations between the two subtypes and immune infiltration in breast cancer, we evaluated the relevance between the two subtypes and 23 kinds of human immune cells using the ssGSEA method. Significant variations were observed in the infiltration of immune cells between the two subtypes (**Figures 3B, C**). The infiltration of activated B cells, CD4^+^ cells, CD8^+^ cells, dendritic cells, CD56 bright natural killer cells, gamma delta T cells, immature B cells, dendritic cells, MDSCs, monocytes, natural killer T cells, natural killer cells, regulatory T cells, T follicular helper cells, type 1 T helper cells, type 17 T helper cells, and type 2 T helper cells was lower in cluster A compared to that in cluster B. The infiltration of mast cells and neutrophils was higher in cluster A. TME score (stromal score, immune score, and estimate score) of the two subtypes were calculated using the ESTIMATE package in R. The level of the stromal score indicates the existence of stromal cells, and the immune score is correlated with the infiltration of immunocytes. Meanwhile, estimate scores represented the aggregation of stromal or immune scores in the TME. Our results revealed that lower TME scores were represented in patients of cluster A (**Figure 3D**). In addition, we investigated the profiles of immune checkpoints and found that most immune checkpoints were differentially expressed in the two groups, including PD-1 (PDCD1), PD-L1 (CD274), and CTLA-4 (**Figure 3E**).

### Identification and validation of the senescence-related signature

**Figure 4:**
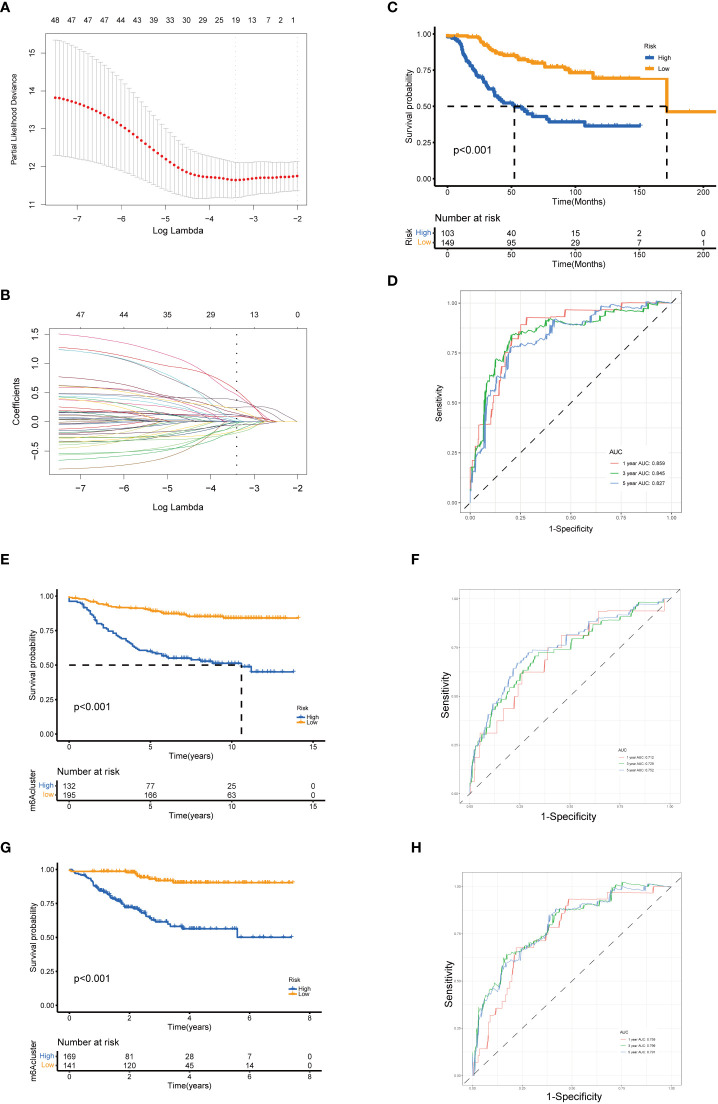
Identification and validation of the senescence-related gene model. **(A)** Tenfold cross-validation for tuning parameter selection in the LASSO model. **(B)** LASSO coefficient profiles of the 19 prognostic genes. A vertical line is drawn at the value chosen by the 10‐fold cross‐validation RFS. Kaplan–Meier curves for the RFS of the two gene subtypes in GSE21653 **(C)**, GSE20685 **(E)**, and GSE25055 **(G)** (log-rank tests, *p* < 0.001). ROC curves to predict the sensitivity and specificity of 1-, 3-, and 5-year survival according to the risk score in GSE21653 **(D)**, GSE20685 **(F)**, and GSE25055 **(H)**. LASSO, least absolute shrinkage and selection operator; RFS, recurrence-free survival.

To establish a predictive model for cancer prognosis, we identified the prognostic genes using univariate Cox regression analysis in the training set (GSE21653). Two hundred and fifty-two patients were classified into high- and low-expression groups according to an optimal cutoff of each gene, and 83 senescence-related genes significantly associated with the RFS were considered as prognostic genes for further analysis. Then, the LASSO-penalized Cox analysis with 10-fold cross-validation was performed to narrow the genes (**Figures 4A, B**). Subsequently, a four-gene-based signature model was developed, consisting of three high-risk genes and one low-risk gene. The risk score of breast cancer patients was calculated using the following formula: Risk score = −0.482 * expression of CPEB1 + 0.468 * expression of NOTCH3 + 0.213 * expression of NUAK1 + 0.321 * expression of PDPK1. Two hundred and fifty-two breast cancer patients from GSE21653 were separated into two groups according to the optimum cutoff score generated using the “survminer” package in R *via* the maximally selected rank statistics. Patients with a score lower than 0.142 belonged to the low-risk group (*n* = 149), whereas those with a risk score higher than 0.142 were placed in the high-risk group (*n* = 103, **Table 2**). The Kaplan–Meier survival curves proved that patients from the training set (GSE21653) with low risk had a significantly favorable RFS compared to patients with high scores (log-rank test, *p* < 0.001; **Figure 4C**). To investigate the prognostic accuracy of this signature, we next performed the time-dependent ROC curve analysis. The areas under the ROC curve (AUC) achieved 0.859, 0.845, and 0.827 at 1, 3, and 5 years of this predictive model (**Figure 4D**). Consistently, we obtained similar results in the validation sets (GSE20685 and GSE25055), indicating that this signature had an extraordinary prognostic accuracy in breast cancer (**Figures 4E–H**).

**Table 2:** Clinicopathologic characteristics of breast cancer patients according to the senescence-related signature.

Variables	GSE21653		*p*-value
	High risk (%)	Low risk (%)	
Age at diagnosis (years)			0.337
≤50	32 (31.1)	55 (36.9)	
>50	71 (68.9)	94 (63.1)	
Tumor size			0.582
T1	19 (18.4)	38 (25.5)	
T2	51 (49.5)	70 (47.0)	
T3	29 (28.2)	37 (24.8)	
Unknown	4 (3.9)	4 (2.7)	
Lymph node status			0.901
Negative	48 (47.1)	68 (46.3)	
Positive	54 (52.9)	79 (53.7)	
Grade			<0.001
I	6 (5.9)	37 (25.3)	
II	24 (23.5)	60 (41.1)	
III	72 (70.6)	49 (33.6)	
ER status			<0.001
Negative	61 (59.2)	49 (33.3)	
Positive	42 (40.8)	98 (66.7)	
PR status			<0.001
Negative	64 (62.1)	60 (40.8)	
Positive	39 (37.9)	87 (59.2)	
HER2 status			0.134
Negative	86 (83.5)	121 (81.2)	
Positive	13 (12.6)	13 (8.7)	
Unknown	4 (3.9)	15 (10.1)	
P53 status			0.003
Wild type	39 (37.9)	86 (57.7)	
Mutant	38 (36.9)	30 (20.1)	
Unknown	26 (25.2)	33 (22.1)	
Ki-67 status			0.011
Negative	14 (13.6)	44 (29.5)	
Positive	67 (65.0)	75 (50.3)	
Unknown	22 (21.4)	30 (20.1)	
Molecular subtype			<0.001
Basal	47 (45.6)	28 (18.8)	
HER2	10 (9.7)	12 (8.1)	
Luminal A	17 (16.5)	68 (45.6)	
Luminal B	23 (22.3)	21 (14.1)	
Normal	6 (5.8)	20 (13.4)	
Vital status			
Alive	53 (51.5)	116 (77.9)	
Dead	50 (48.5)	33 (22.1)	
Median follow-up (months)			

ER, estrogen receptor; PR, progesterone receptor; HER2, human epidermal growth factor receptor 2; P53, tumor protein P53; Ki-67, proliferation marker protein Ki-67.

**Figure 5:**
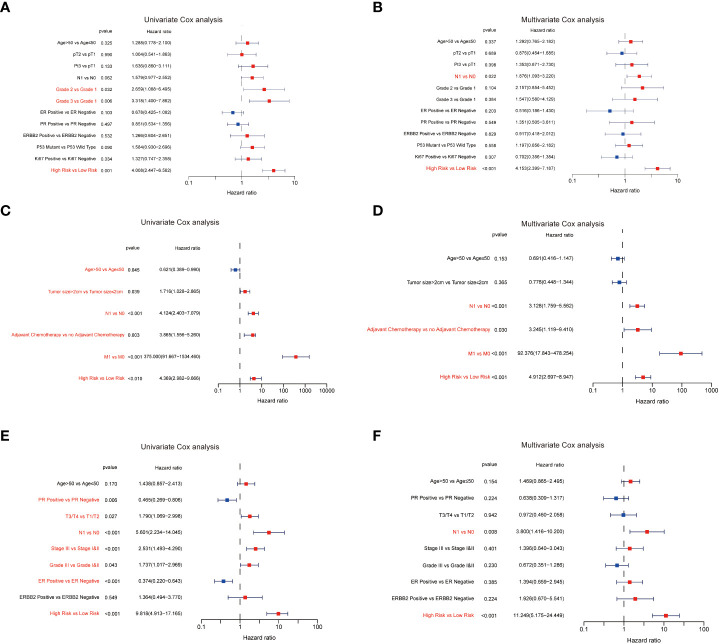
The predictive value of the senescence‐related signature. The results of the univariate Cox analysis in GSE21653 **(A)**, GSE20685 **(C)**, and GSE25055 **(E)**. The results of the multivariate Cox analysis in GSE21653 **(B)**, GSE20685 **(D)**, and GSE25055 **(F)**.

**Figure 6:**
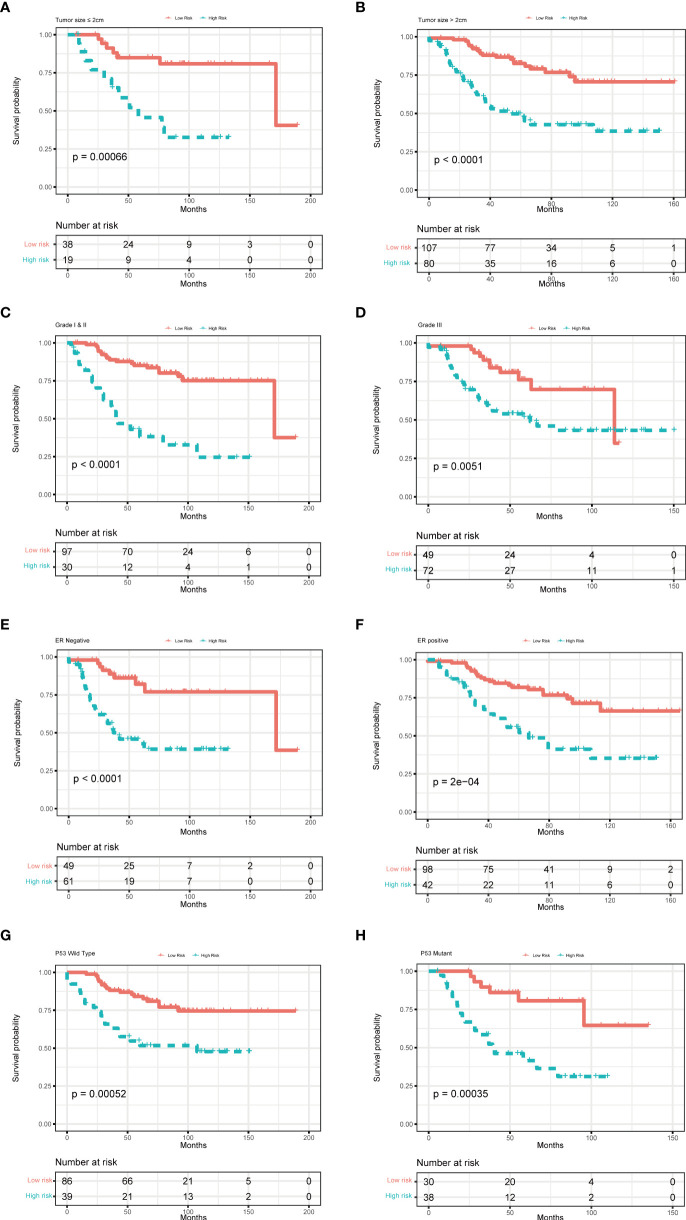
Multivariate Cox analysis for patients according to the predictive model stratified by clinicopathological risk factors. **(A, B)** tumor size, **(C, D)** tumor grade, **(E, F)** ER status, and **(G, H)** P53 mutation.

To further examine the predictive value of this senescence-related signature, univariate and multivariate Cox proportional hazards regression analyses were performed in the GSE21653, GSE20685, and GSE25055 datasets. Our findings demonstrated that the senescence‐related signature was an independent risk factor when controlling for the classical clinicopathologic factors (**Figure 5**). When we separated the patients by clinical risk factors, including tumor size, grade, ER status, and p53 mutation, the senescence-related signature was still a gainful prognostic model (**Figure 6**).

### Relationship between the risk model and TME

**Figure 7:**
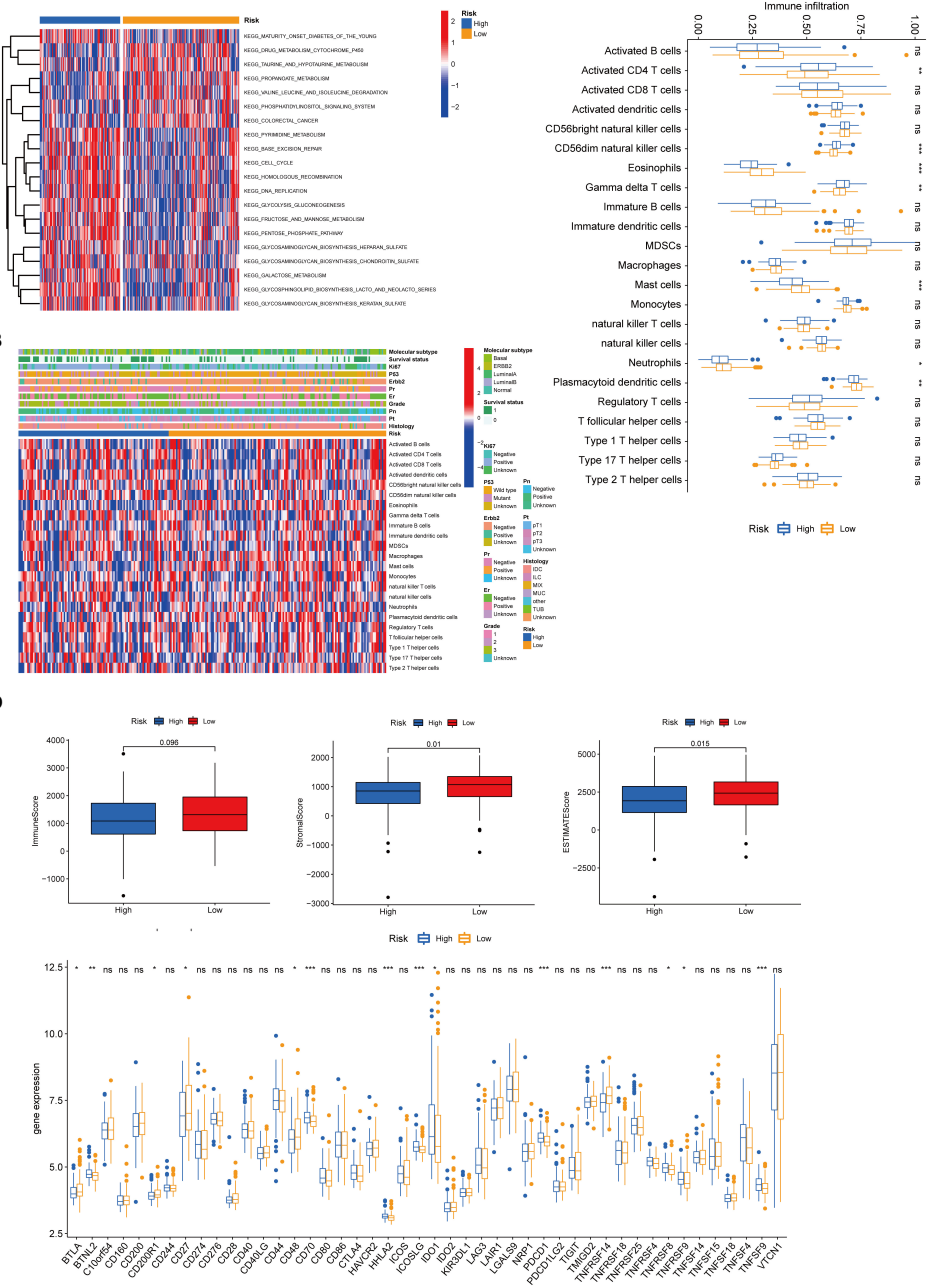
Correlations of the tumor immune cell microenvironment and risk score. **(A)** GSVA of the biological pathways between the two risk groups, in which red and blue represent activated and inhibited pathways, respectively. **(B)** Heatmap of the tumor-infiltrating cell in the two risk groups. **(C)** The abundance of the 23 infiltrating immune cell types in the two risk groups. **(D)** Correlations between the two subtypes and TME score. **(E)** Differences in the expression of checkpoints in the two risk groups. BRCA, breast cancer; GSVA, gene set variation analysis; TME, tumor microenvironment. *P value < 0.05; **P value < 0.01; ***P value < 0.001.

GSVA enrichment analysis showed that cell cycle and DNA replication pathways were positively correlated to high-risk scores. At the same time, drug metabolism cytochrome and taurine and hypotaurine metabolism were positively relevant with low-risk scores (**Figure 7A**). GSEA analysis indicated that biological functions related to cellular senescence and aging were significantly enriched in the high-risk group (**Supplementary Figure 2B**). We further explored the relationships between risk score and infiltration of immune cells of breast cancer using the ESTIMATE package in R (**Figure 7B**). The infiltration levels of activated CD4^+^ T cells, CD56dim natural killer cells, and gamma delta T cells in the high-risk group were significantly higher compared to those in the low-risk group. Nevertheless, the infiltration of eosinophils, mast cells, neutrophils, and plasmacytoid dendritic cells was significantly decreased in the high-risk group (**Figure 7C**). We also calculated the TME scores of the high- and low-risk groups, and our results demonstrated higher stromal and ESTIMATE scores for patients with low risk (**Figure 7D**). We then investigated the expression of immune checkpoints in the two groups, and it was found that the expression of PD-1 (PDCD1) was differentially expressed in the two groups (**Figure 7E**). We further used GSE25055 to determine whether this signature was associated with treatment outcomes. It was found that a lower risk score was associated with a higher pathological response rate in patients given neoadjuvant chemotherapy (**Supplementary Figure 3**).

### Analysis of the four senescence-related genes used for the prognostic signature

**Figure 8:**
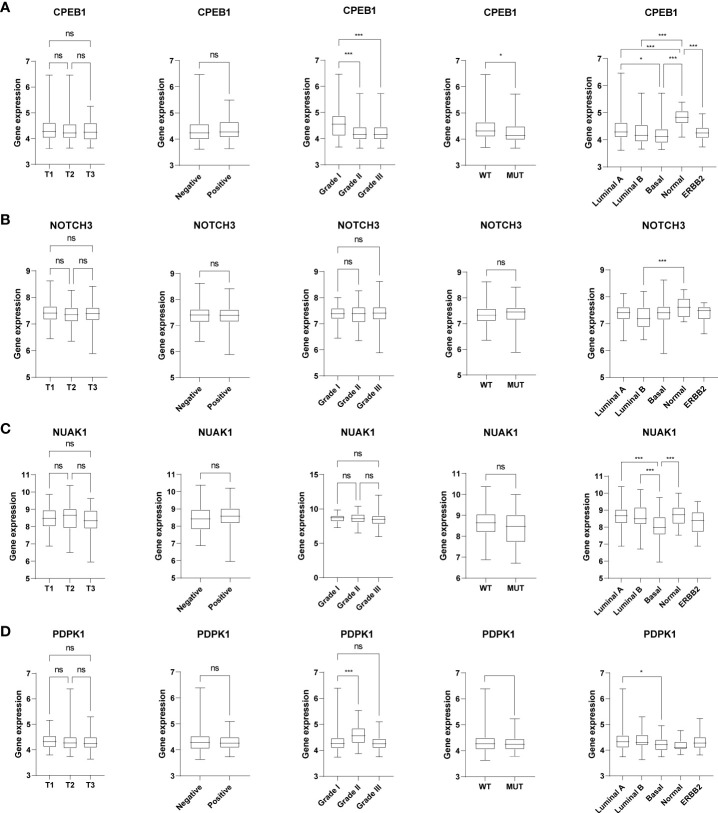
The relationships between the four senescence-related genes and clinical features. The box plots showed the correlations between *CPEB1* **(A)**, *NOTCH3* **(B)**, *NUAK1* **(C)**, and *PDPK1* **(D)** and tumor stage, ER status, tumor grade, P53 mutation, and molecular subtypes of BRCA. *P value < 0.05; ***P value < 0.001.

**Figure 9:**
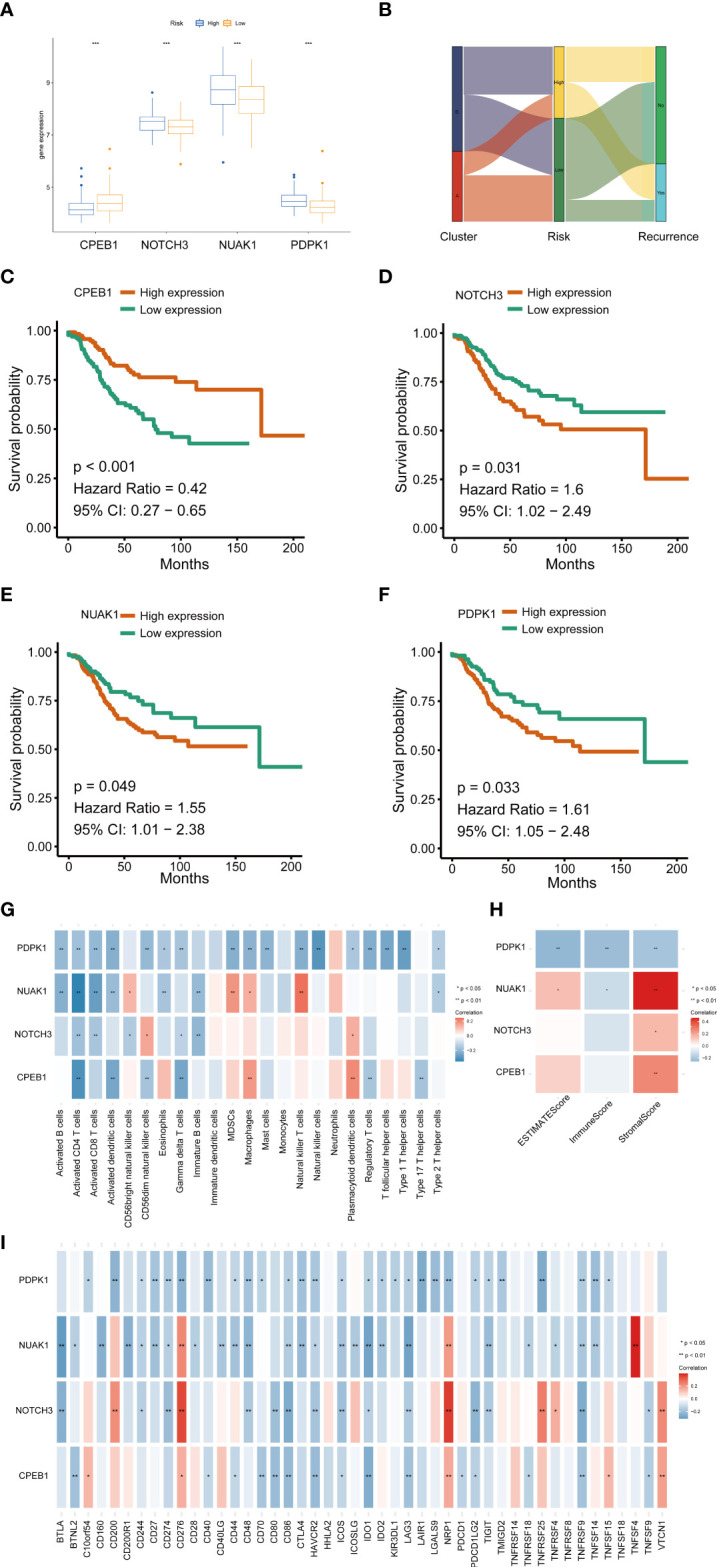
The relationships between the four senescence-related genes and prognostics and the correlations of the tumor immune cell microenvironment and the four senescence-related genes. **(A)** The expression of the four prognostic genes. **(B)** Sankey diagram. **(C–F)** Univariate Cox regression analysis of the four prognostic genes in the signature. **(G)** The correlation between the four genes and activated immune cells. **(H)** The relationship between the four genes and TME score. **(I)** The correlation between the four genes and immune checkpoint. *P value < 0.05; **P value < 0.01; ***P value < 0.001.

**Figure 10:**
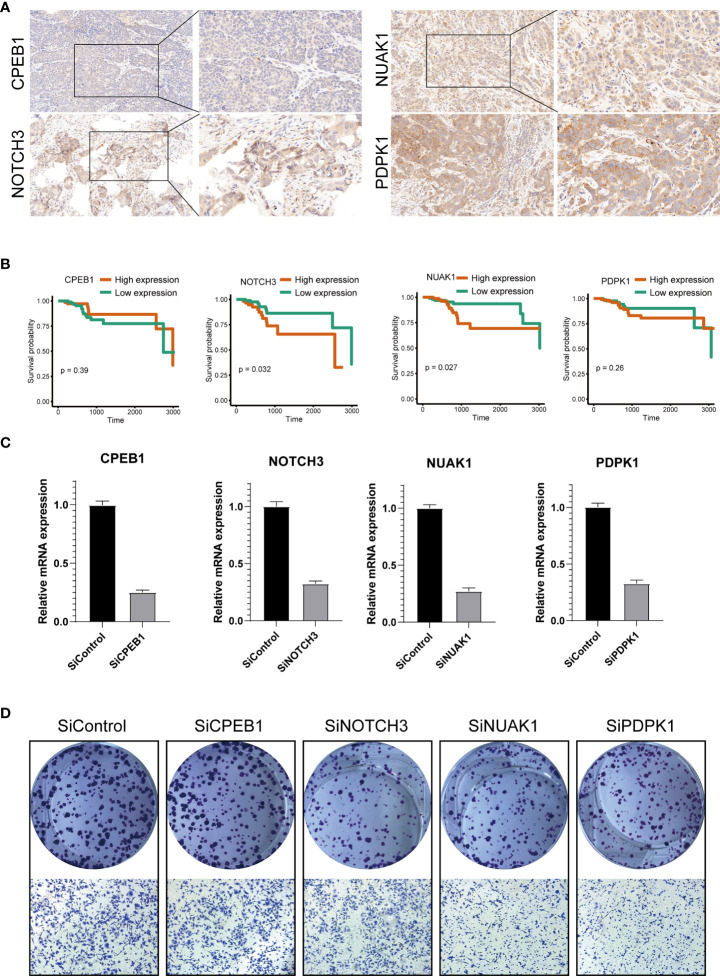
Validation of the four prognostic genes by functional analysis. **(A)** Immunohistochemistry analysis of *CPEB1*, *NOTCH3*, *NUAK1*, and *PDPK1* in breast cancer tissues. **(B)** The high expression levels of *NOTCH3* and *NUAK1* were associated with a poor prognosis. The Cox proportion hazards model was used to understand the significance between the two groups. Tissue microarray was obtained from Alenabio Technology Co., Ltd., Xian, China. The tissue microarray contained 138 breast cancer specimens. **(C)** siRNA knockdown efficiencies. **(D)** The colony formation and migration analysis of breast cancer cell depletion with *CPEB1*, *NOTCH3*, *NUAK1*, and *PDPK1*.

We further explored the expression levels of the four prognostic genes in breast cancer patients (**Figure 8**). The results demonstrated that the expression level of *CPEB1* was significantly decreased in grades II and III compared to that in grade I. Meanwhile, the expression of *CPEB1* was negatively correlated with P53 mutation. In addition, the expression levels of *CPEB1*, *NOTCH3*, *NUAK1*, and *PDPK1* were significantly discrepant among the molecular subtypes of breast cancer. Consistently, *CPEB1* was upregulated in the low-risk group, while *NOTCH3*, *NUAK1*, and *PDPK1* were overexpressed in the high-risk group (**Figure 9A**). The Sankey analysis indicated that over half of the patients in cluster A were grouped into high risk (**Figure 9B**). The Kaplan–Meier survival curves indicated a longer RFS in patients with a high-expression level of *CPEB1* or low-expression levels of *NOTCH3*, *NUAK1*, and *PDPK1* (**Figures 9C–F**). We further assessed the relationship between these four genes and the abundance of immune cells. It was observed that the expression of *PDK1* was negatively correlated with most immune cells, while *NUAK1*, *NOTCH3*, and *CPEB1* were positively related to several immune cells, including MDSCs, macrophages, and plasmacytoid dendritic cells (**Figure 9G**). We next evaluated the correlations between TME scores and the expression of the four genes, indicating that the TME scores were negatively associated with *PDPK1* but positively associated with *NUAK1*, *NOTCH3*, and *CPEB1* (**Figure 9H**). In addition, we investigated the associations between the expression of immune checkpoints and the four senescence-related genes. **Figure 9I** shows that a large proportion of the 46 immune checkpoints were negatively associated with the expression of *PDPK1*. Several markers were positively associated with the expression of *NUAK1*, *NOTCH3*, and *CPEB1*, including CD276 and NRP1. Meanwhile, it is worth mentioning that the expression of CD274 had a significantly negative correlation with *PDPK1*, *NUAK1*, and *NOTCH3*. To further confirm the protein expression and the prognostic value of the four genes, we performed immunohistochemistry (IHC) analysis using TMA which contained 138 patients. The results of the IHC analysis demonstrated that *NOTCH3*, *NUAK1*, and *PDPK1* were highly expressed in breast cancer tissues (**Figure 10A**). The survival analysis indicated that high NOTCH3 and NUAK1 protein levels were associated with a poor prognosis (**Figure 10B**).

We silenced the expression of each gene through siRNA (**Figure 10C**) to further verify the biological functions of *CPEB1*, *NOTCH3*, *NUAK1*, and *PDPK1*. The results of colony formation indicated that depletion of *NOTCH3*, *NUAK1*, or *PDPK1* inhibited the colony formation ability of MDA-MB-231 cells, while no significant difference was observed upon *CPEB1* depletion. Consistently, depletion of *NOTCH3*, *NUAK1*, or *PDPK1* decreased cancer cell migration (**Figure 10D**).

## Discussion

Numerous studies have revealed that senescence can modulate the progression of breast cancer and can interact with therapies, both potentially being induced by treatment and influencing treatment resistance. Since senescence is considered as a tumor suppressor mechanism, induction of cancer cell senescence is the focus of research into novel tumor treatments. In the present study, we constructed a senescence-related signature to predict the prognosis of breast cancer and used GSE25055 to determine whether this signature was associated with treatment outcomes. We observed that the signature was positively associated with the pathological response. Higher risk scores indicated poor pathological response to neoadjuvant chemotherapy. It was reported that radiation could induce senescence in breast cancer, and these cells in turn released SASPs to promote the migration and invasion of neighboring cancer cells (23). Palbociclib (a CDK4/6 inhibitor) exerts antiproliferative effects on breast cancer cells and induces senescence and cell cycle arrest (24). In addition, some chemotherapy drugs can induce senescence of breast cancer as well. Breast cancer cells exposed to doxorubicin undergo widespread senescence (25). In MMTV-Wnt1 mouse models of breast carcinoma, doxorubicin induces senescence and the expression of the SASP factors. These cytokines produced by senescent cells could promote the proliferation of surrounding non-senescent cancer cells and lead to clinical relapse (26). A previous study explored the relationships between SASP positivity and tumor microenvironments in invasive breast cancer (IBC) tissues. SASP positivity is associated with a poor prognosis in luminal A IBC, while SASP-positive TNBC indicates better survival. The multivariate analysis demonstrates that SASP positivity is an independent prognostic factor in both luminal A IBC and TNBC (27). Nevertheless, most studies only focus on a single gene or a single type of immune cell. Our research revealed an overall profile of senescence-related genes and TME in breast cancer. Firstly, we separated breast cancer patients into two clusters (cluster A and cluster B) based on the expression of senescence‐related genes. Patients in cluster A represented a higher level of senescence as revealed by the GSEA analysis and exhibited a better prognosis. Patients in cluster B tended to have tumors with advanced stage, and there is a dramatic preponderance of TNBC in cluster B. These would be expected to produce at least the survival difference. To clarify the infiltration of immune cells and activated pathways in breast cancer, we next investigated the TME in the two clusters, showing that cluster A negatively correlates with immune activation and infiltration. Noting that cluster B tumors are often TNBC or HER2-positive, it is not a surprise that immune pathways are more enriched in cluster B, as immune infiltrates are significantly more common in these tumors. We further constructed a predictive signature of senescence-related genes using high-throughput expression profiles, and patients were divided into low- or high-risk groups. Patients in the low-risk group represented a higher level of senescence and exhibited better prognosis. The results of the ROC analysis in the training and validation sets demonstrated that this signature exhibited good diagnostic efficiency for the 1-, 3-, and 5-year disease-relapse events. Furthermore, we investigated the relationship between this model and TME, and the results indicated that *CPEB1*, *NOTCH3*, *NUAK1*, and *PDPK1* were strongly associated with the expression of tumor checkpoints and tumor immune infiltration.

The biological functions of senescence-related genes have been studied previously. *CPEB1* is a post-transcriptional regulatory factor regulating mRNA translation by dynamically adjusting poly (A) tail length (28). The prognostics of tumor patients were affected by the level of *CPEB1*. Nagaoka et al. revealed that the low expression of *CPEB1* promoted epithelial-to-mesenchymal transition and metastasis in breast cancer (29). Interestingly, these malignant phenotypes could be accelerated by estrogen in breast cancer (30). Previous studies also demonstrated that the low level of *CPEB1* was linked to increased metastasis and angiogenesis in gastric cancer (GC), while *CPEB1* boosted ferroptosis by inhibiting TWIST1 (31). Meanwhile, it was demonstrated that the negative regulation between *CPEB1* and SIRT1 suppressed HCC stemness (32).

Notch proteins are cell membrane receptors crucial for cell communication (33). Abnormal Notch signaling activation promotes cancer progression, cancer stem cell activation, and tumor chemoresistance (34). For instance, it was shown that Notch3 accelerated the development of prostate cancer-induced bone lesions through MMP-3 (35). Meanwhile, previous research proved that breast cancer tumorigenesis could be regulated by phosphorylation of Notch3, which might be affected by PTEN transactivation (36–38). Furthermore, many molecules can also affect tumorigenesis by influencing the Notch3 pathway. For example, Hsa_circ_0058124 facilitated papillary thyroid cancer progression and invasiveness *via* the NOTCH3/GATAD2A axis (39).

NUAK family kinase 1 (NUAK1) was reported to play a role in oncogenesis by the improvement of glycolysis (40), induction of ferroptosis (41), and phosphorylation of downstream molecules (42). However, NUAK could prevent tumorigenesis progression due to its protective function from oxidative stress in colorectal tumors (43). Through targeting the NUAK1 kinase, miR-622 inhibited the motility phenotype of breast cancer (44). However, the specific mechanisms of NUAK1 in breast cancer progression are unclear and need further exploration.

PDPK1 is a phosphorylation-regulated kinase that is essential for numerous signaling pathway activation and cellular processes (45). PDPK1 proved to be an intriguing molecular target in physiological and pathological activities *via* promotion of CD8^+^ T cells (46), regulation of autophagy (45), the resistance of chemotherapy (47), phosphorylation of the kinase family (48), and activation of other downstream pathways. It was demonstrated that PDPK1 regulates prostate cancer cell survival *via* SGK3 (49). Meanwhile, PDPK1 was regulated by several molecular pathways, including mTORC2/PI3K, and then facilitated the proliferation of breast cancer cells (50). In a word, the senescence-related genes play a crucial part in the tumorigenesis of breast cancer, where deeper studies are required to probe their functions and mechanisms.

In this research, we established a prognostic model based on four senescence-related genes to predict the RFS of breast cancer patients. This model provided a beneficial pattern for clinical outcomes analysis of breast cancer patients. Our study had several notable limitations, although our model demonstrated an accurate forecasting ability. Firstly, the sample sizes of our investigation were finite, and large-scale cohort studies were imperative for judging the value of this model. Secondly, the relationship between these senescence-related genes and other meaningful biomarkers, such as BRCA1, BRCA2, and HER-2, was unclear. Thirdly, as these are bulk RNA signatures such that it is not known if senescence genes are expressed in cancer or stroma and as the immune cell identification process is again bulk and so has a lot of surrogacy, validation with actual tissue to confirm that functional protein levels match their RNA signatures and confirming that immune infiltrates match the RNA predictions are important to take this work further. Furthermore, specific *in-vivo* and *in-vitro* experiments are required to verify the function of these senescence-related genes.

In summary, our study established a novel classification for breast cancer based on the mRNA expression profiles of cellular senescence-related genes. We observed that patients’ survival, clinicopathologic features, and immune status were significantly different between the two clusters. We also developed a senescence scoring system to predict the RFS of patients with breast cancer, which proved to be a beneficial tool for predicting the clinical outcomes of breast cancer patients.

## Data availability statement

The original contributions presented in the study are included in the article/**Supplementary Material**. Further inquiries can be directed to the corresponding author.

## Author contributions

YZ, JT made substantial contributions to the design of the work, acquisition, analysis and interpretation of data for the work and drafted the work. LX and LZ revised it critically for important intellectual content. JT, SL, YT, provide approval for publication of the content. GL, JC agree to be accountable for all aspects of the work in ensuring that questions related to the accuracy or integrity of any part of the work are appropriately investigated and resolved. All authors contributed to the article and approved the submitted version.

## Funding

This work was supported by the Hunan Provincial Natural Science Foundation of China (202JJ40815).

## Conflict of interest

The reviewer YZ declared a shared parent affiliation with the authors to the handling editor at the time of the review.

The remaining authors declare that the research was conducted in the absence of any commercial or financial relationships that could be construed as a potential conflict of interest.

## Publisher’s note

All claims expressed in this article are solely those of the authors and do not necessarily represent those of their affiliated organizations, or those of the publisher, the editors and the reviewers. Any product that may be evaluated in this article, or claim that may be made by its manufacturer, is not guaranteed or endorsed by the publisher.

## Supplementary material

Supplementary Figure 1 Multivariate Cox analysis for patients according to the senescence-related subtype stratified by clinicopathological risk factors. **(A, B)** tumor size, **(C, D)** tumor grade, **(E, F)** Tipple-negative breast cancer.

Supplementary Figure 2 Gene set enrichment analysis (GSEA). **(A)** Cellular senescence and aging were significantly enriched in Cluster A. **(B)** Cellular senescence and aging were significantly enriched in high risk group.

Supplementary Figure 3 Relationships between the senescence-related score and pathological response to neoadjuvant chemotherapy.

## Abbreviations

ROC, receiver operating characteristic curve; ER, estrogen receptor; PR, progesterone receptor; TNBC, triple-negative breast cancer; DDR, DNA damage response; CDKi, cyclin-dependent kinase inhibitors; PM, plasma membrane; TME, tumor microenvironment; RFS, recurrence-free survival; GEO, Gene Expression Omnibus; CDF, cumulative distribution function; GO, Gene Ontology; KEGG, Kyoto Encyclopedia of Genes and Genomes; GSVA, gene set variation analysis; LASSO, least absolute shrinkage and selection operator; GC, gastric cancer.

## References

[1] Sung H Ferlay J Siegel RL Laversanne M Soerjomataram I Jemal A. Global cancer statistics 2020: GLOBOCAN estimates of incidence and mortality worldwide for 36 cancers in 185 countries. CA Cancer J Clin (2021) 71(3):209–49. doi: 10.3322/caac.21660 33538338

[2] Britt KL Cuzick J Phillips KA. Key steps for effective breast cancer prevention. Nat Rev Cancer (2020) 20(8):417–36. doi: 10.1038/s41568-020-0266-x 32528185

[3] Regan MM Pagani O Francis PA Fleming GF Walley BA Kammler R. Predictive value and clinical utility of centrally assessed ER, PgR, and ki-67 to select adjuvant endocrine therapy for premenopausal women with hormone receptor-positive, HER2-negative early breast cancer: TEXT and SOFT trials. Breast Cancer Res Treat (2015) 154(2):275–86. doi: 10.1007/s10549-015-3612-z PMC4749471 26493064

[4] Hernandez-Segura A Nehme J Demaria M. Hallmarks of cellular senescence. Trends Cell Biol (2018) 28(6):436–53. doi: 10.1016/j.tcb.2018.02.001 29477613

[5] He S Sharpless NE. Senescence in health and disease. Cell (2017) 169(6):1000–11. doi: 10.1016/j.cell.2017.05.015 PMC5643029 28575665

[6] Sharpless NE Sherr CJ. Forging a signature of *in vivo* senescence. Nat Rev Cancer (2015) 15(7):397–408. doi: 10.1038/nrc3960 26105537

[7] Birch J Gil J. Senescence and the SASP: many therapeutic avenues. Genes Dev (2020) 34(23-24):1565–76. doi: 10.1101/gad.343129.120 PMC7706700 33262144

[8] Herranz N Gil J. Mechanisms and functions of cellular senescence. J Clin Invest (2018) 128(4):1238–46. doi: 10.1172/JCI95148 PMC5873888 29608137

[9] Basisty N Kale A Jeon OH Kuehnemann C Payne T Rao C. A proteomic atlas of senescence-associated secretomes for aging biomarker development. PloS Biol (2020) 18(1):e3000599. doi: 10.1371/journal.pbio.3000599 31945054 PMC6964821

[10] Coppé JP Patil CK Rodier F Sun Y Muñoz DP Goldstein J. Senescence-associated secretory phenotypes reveal cell-nonautonomous functions of oncogenic RAS and the p53 tumor suppressor. PloS Biol (2008) 6(12):2853–68. doi: 10.1371/journal.pbio.0060301 PMC2592359 19053174

[11] Calcinotto A Kohli J Zagato E Pellegrini L Demaria M Alimonti A. Cellular senescence: Aging, cancer, and injury. Physiol Rev (2019) 99(2):1047–78. doi: 10.1152/physrev.00020.2018 30648461

[12] Avena P Anselmo W Whitaker-Menezes D Wang C Pestell RG Lamb RS. Compartment-specific activation of PPARγ governs breast cancer tumor growth, *via* metabolic reprogramming and symbiosis. Cell Cycle (2013) 12(9):1360–70. doi: 10.4161/cc.24289 PMC3674064 23574724

[13] Caldwell ME DeNicola GM Martins CP Jacobetz MA Maitra A Hruban RH. Cellular features of senescence during the evolution of human and murine ductal pancreatic cancer. Oncogene (2012) 31(12):1599–608. doi: 10.1038/onc.2011.350 PMC3397306 21860420

[14] Liao EC Hsu YT Chuah QY Lee YJ Hu JY Huang TC. Radiation induces senescence and a bystander effect through metabolic alterations. Cell Death Dis (2014) 5(5):e1255. doi: 10.1038/cddis.2014.220 24853433 PMC4047910

[15] Coppé JP Desprez PY Krtolica A Campisi J. The senescence-associated secretory phenotype: the dark side of tumor suppression. Annu Rev Pathol (2010) 5:99–118. doi: 10.1146/annurev-pathol-121808-102144 20078217 PMC4166495

[16] Hinshaw DC Shevde LA. The tumor microenvironment innately modulates cancer progression. Cancer Res (2019) 79(18):4557–66. doi: 10.1158/0008-5472.CAN-18-3962 PMC6744958 31350295

[17] Wu T Dai Y. Tumor microenvironment and therapeutic response. Cancer Lett (2017) 387:61–8. doi: 10.1016/j.canlet.2016.01.043 26845449

[18] Prata L Ovsyannikova IG Tchkonia T Kirkland JL. Senescent cell clearance by the immune system: Emerging therapeutic opportunities. Semin Immunol (2018) 40:101275. doi: 10.1016/j.smim.2019.04.003 31088710 PMC7061456

[19] Li L Liu X Sanders KL Edwards JL Ye J Si F. TLR8-mediated metabolic control of human treg function: A mechanistic target for cancer immunotherapy. Cell Metab (2019) 29(1):103–23.e5. doi: 10.1016/j.cmet.2018.09.020 30344014 PMC7050437

[20] Liu X Mo W Ye J Li L Zhang Y Hsueh EC. Regulatory T cells trigger effector T cell DNA damage and senescence caused by metabolic competition. Nat Commun (2018) 9(1):249. doi: 10.1038/s41467-017-02689-5 29339767 PMC5770447

[21] Shen J Song R Fuemmeler BF McGuire KP Chow WH Zhao H. Biological aging marker p16(INK4a) in T cells and breast cancer risk. Cancers (Basel) (2020) 12(11). doi: 10.3390/cancers12113122 PMC7692397 33114473

[22] Gu P Chen X Xie R Xie W Huang L Dong W. A novel AR translational regulator lncRNA LBCS inhibits castration resistance of prostate cancer. Mol Cancer (2019) 18(1):109. doi: 10.1186/s12943-019-1037-8 31221168 PMC6585145

[23] Yu YC Yang PM Chuah QY Huang YH Peng CW Lee YJ. Radiation-induced senescence in securin-deficient cancer cells promotes cell invasion involving the IL-6/STAT3 and PDGF-BB/PDGFR pathways. Sci Rep (2013) 3:1675. doi: 10.1038/srep01675 23591770 PMC3628221

[24] Lee CS Baek J Han SY. The role of kinase modulators in cellular senescence for use in cancer treatment. Molecules (2017) 22(9). doi: 10.3390/molecules22091411 PMC6151769 28841181

[25] Pham TH Park HM Kim J Hong JT Yoon DY. Interleukin-32θ triggers cellular senescence and reduces sensitivity to doxorubicin-mediated cytotoxicity in MDA-MB-231 cells. Int J Mol Sci (2021) 22(9). doi: 10.3390/ijms22094974 PMC8124300 34067074

[26] Jackson JG Pant V Li Q Chang LL Quintás-Cardama A Garza D. p53-mediated senescence impairs the apoptotic response to chemotherapy and clinical outcome in breast cancer. Cancer Cell (2012) 21(6):793–806. doi: 10.1016/j.ccr.2012.04.027 22698404 PMC3376352

[27] Park MH Choi JE Kim JR Bae YK. Immunohistochemical expressions of senescence-associated secretory phenotype and its association with immune microenvironments and clinicopathological factors in invasive breast cancer. Pathol Oncol Res (2021) 27:1609795. doi: 10.3389/pore.2021.1609795 34267603 PMC8276694

[28] D’Ambrogio A Nagaoka K Richter JD. Translational control of cell growth and malignancy by the CPEBs. Nat Rev Cancer (2013) 13(4):283–90. doi: 10.1038/nrc3485 23446545

[29] Nagaoka K Fujii K Zhang H Usuda K Watanabe G Ivshina M. CPEB1 mediates epithelial-to-mesenchyme transition and breast cancer metastasis. Oncogene (2016) 35(22):2893–901. doi: 10.1038/onc.2015.350 PMC4809797 26411364

[30] Sovijit W Sovijit W Ishii Y Kambe J Fujita T Watanabe G. Estrogen promotes increased breast cancer cell proliferation and migration through downregulation of CPEB1 expression. Biochem Biophys Res Commun (2021) 534:871–6. doi: 10.1016/j.bbrc.2020.10.085 33162033

[31] Wang J Wang T Zhang Y Liu J Song J Han Y. CPEB1 enhances erastin-induced ferroptosis in gastric cancer cells by suppressing twist1 expression. IUBMB Life (2021) 73(9):1180–90. doi: 10.1002/iub.2525 34184391

[32] Xu M Fang S Song J Chen M Zhang Q Weng Q. CPEB1 mediates hepatocellular carcinoma cancer stemness and chemoresistance. Cell Death Dis (2018) 9(10):957. doi: 10.1038/s41419-018-0974-2 30237545 PMC6148052

[33] Artavanis-Tsakonas S Rand MD Lake RJ. Notch signaling: cell fate control and signal integration in development. Science (1999) 284(5415):770–6. doi: 10.1126/science.284.5415.770 10221902

[34] Xiu M Wang Y Li B Wang X Xiao F Chen S. The role of Notch3 signaling in cancer stemness and chemoresistance: Molecular mechanisms and targeting strategies. Front Mol Biosci (2021) 8:694141. doi: 10.3389/fmolb.2021.694141 34195229 PMC8237348

[35] Ganguly SS Hostetter G Tang L Frank SB Saboda K Mehra R. Notch3 promotes prostate cancer-induced bone lesion development *via* MMP-3. Oncogene (2020) 39(1):204–18. doi: 10.1038/s41388-019-0977-1 PMC6938550 31467432

[36] Landor SKJ Santio NM Eccleshall WB Paramonov VM Gagliani EK Hall D. PIM-induced phosphorylation of Notch3 promotes breast cancer tumorigenicity in a CSL-independent fashion. J Biol Chem (2021) 296:100593. doi: 10.1016/j.jbc.2021.100593 33775697 PMC8100066

[37] Zhang YQ Liang YK Wu Y Chen M Chen WL Li RH. Notch3 inhibits cell proliferation and tumorigenesis and predicts better prognosis in breast cancer through transactivating PTEN. Cell Death Dis (2021) 12(6):502. doi: 10.1038/s41419-021-03735-3 34006834 PMC8131382

[38] Leontovich AA Jalalirad M Salisbury JL Mills L Haddox C Schroeder M. NOTCH3 expression is linked to breast cancer seeding and distant metastasis. Breast Cancer Res (2018) 20(1):105. doi: 10.1186/s13058-018-1020-0 30180881 PMC6123953

[39] Yao Y Chen X Yang H Chen W Qian Y Yan Z. Hsa_circ_0058124 promotes papillary thyroid cancer tumorigenesis and invasiveness through the NOTCH3/GATAD2A axis. J Exp Clin Cancer Res (2019) 38(1):318. doi: 10.1186/s13046-019-1321-x 31324198 PMC6642504

[40] Escalona E Muñoz M Pincheira R Elorza ÁA Castro AF. Cytosolic NUAK1 enhances ATP production by maintaining proper glycolysis and mitochondrial function in cancer cells. Front Oncol (2020) 10:1123. doi: 10.3389/fonc.2020.01123 32754444 PMC7367139

[41] Yang C Zhang Y Lin S Liu Y Li W. Correction for: Suppressing the KIF20A/NUAK1/Nrf2/GPX4 signaling pathway induces ferroptosis and enhances the sensitivity of colorectal cancer to oxaliplatin. Aging (Albany NY) (2021) 13(14):19077. doi: 10.18632/aging.203382 34314378 PMC8351681

[42] Cossa G Roeschert I Prinz F Baluapuri A Vidal RS Schülein-Völk C. Localized inhibition of protein phosphatase 1 by NUAK1 promotes spliceosome activity and reveals a MYC-sensitive feedback control of transcription. Mol Cell (2021) 81(11):2495. doi: 10.1016/j.molcel.2021.05.013 34087181 PMC8189434

[43] Port J Muthalagu N Raja M Ceteci F Monteverde T Kruspig B. Colorectal tumors require NUAK1 for protection from oxidative stress. Cancer Discovery (2018) 8(5):632–47. doi: 10.1158/2159-8290.CD-17-0533 PMC5935231 29500295

[44] Orlandella FM Mariniello RM Mirabelli P De Stefano AE Iervolino PLC Lasorsa VA. miR-622 is a novel potential biomarker of breast carcinoma and impairs motility of breast cancer cells through targeting NUAK1 kinase. Br J Cancer (2020) 123(3):426–37. doi: 10.1038/s41416-020-0884-9 PMC7403386 32418991

[45] Hu B Zhang Y Deng T Gu J Liu J Yang H. PDPK1 regulates autophagosome biogenesis by binding to PIK3C3. Autophagy (2021) 17(9):2166–83. doi: 10.1080/15548627.2020.1817279 PMC8496706 32876514

[46] Fan L Zhang CJ Zhu L Chen J Zhang Z Liu P. FasL-PDPK1 pathway promotes the cytotoxicity of CD8(+) T cells during ischemic stroke. Transl Stroke Res (2020) 11(4):747–61. doi: 10.1007/s12975-019-00749-0 32036560

[47] Li D Mullinax JE Aiken T Xin H Wiegand G Anderson A. Loss of PDPK1 abrogates resistance to gemcitabine in label-retaining pancreatic cancer cells. BMC Cancer (2018) 18(1):772. doi: 10.1186/s12885-018-4690-1 30064387 PMC6069886

[48] Gagliardi PA Puliafito A Primo L. PDK1: At the crossroad of cancer signaling pathways. Semin Cancer Biol (2018) 48:27–35. doi: 10.1016/j.semcancer.2017.04.014 28473254

[49] Nalairndran G Hassan Abdul Razack A Mai CW Fei-Lei Chung F Chan KK Hii LW. Phosphoinositide-dependent kinase-1 (PDPK1) regulates serum/glucocorticoid-regulated kinase 3 (SGK3) for prostate cancer cell survival. J Cell Mol Med (2020) 24(20):12188–98. doi: 10.1111/jcmm.15876 PMC7578863 32926495

[50] Mangé A Coyaud E Desmetz C Laurent E Béganton B Coopman P. FKBP4 connects mTORC2 and PI3K to activate the PDK1/Akt-dependent cell proliferation signaling in breast cancer. Theranostics (2019) 9(23):7003–15. doi: 10.7150/thno.35561 PMC6815969 31660083

