# Supplementary figures and images for: Identification of senescence-related subtypes, establishment of a prognosis model, and characterization of a tumor microenvironment infiltration in breast cancer
Source: Front Immunol. 2022 Aug 22;13:921182. doi: 10.3389/fimmu.2022.921182 (PMC9441960; doi:10.3389/fimmu.2022.921182)

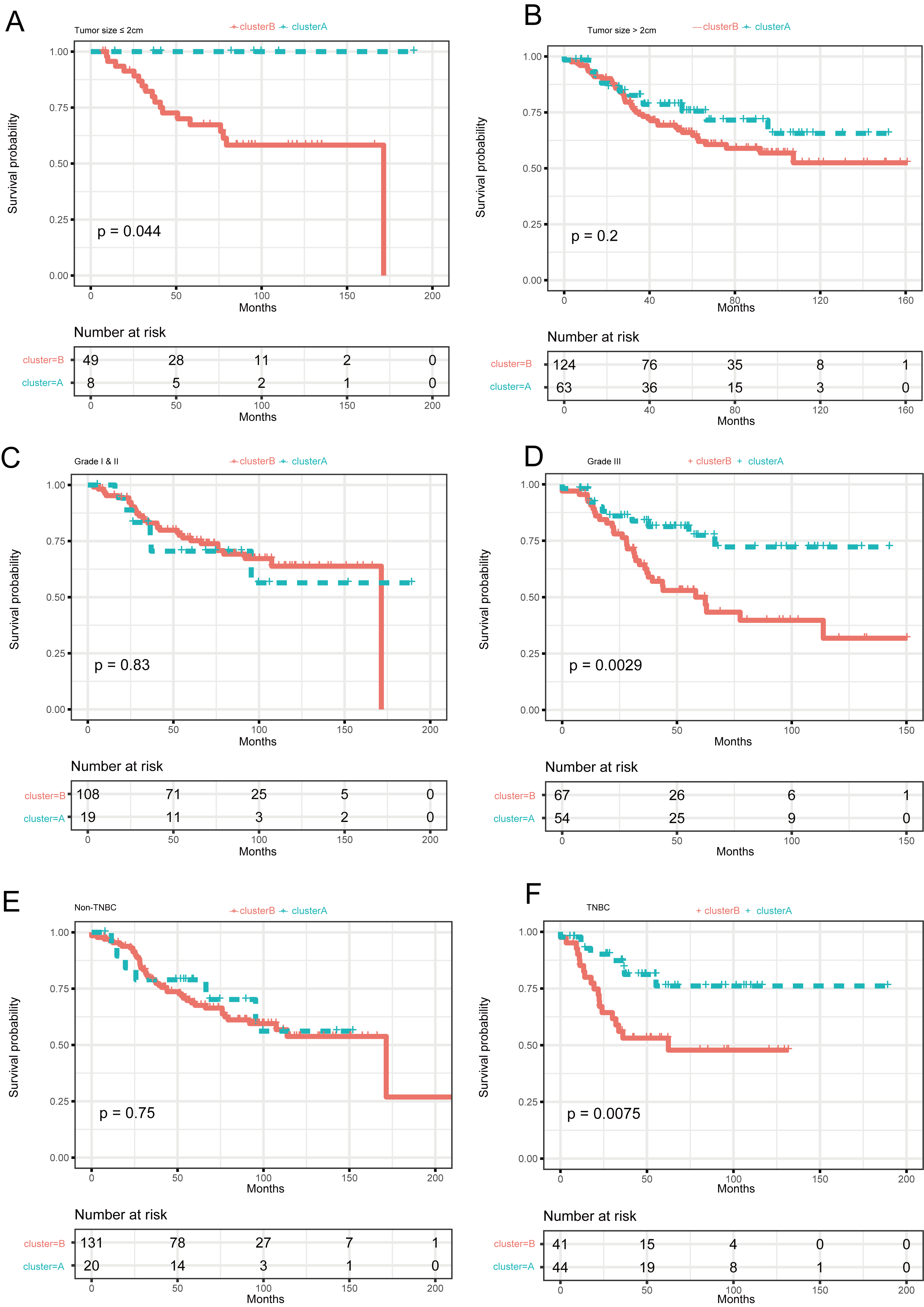

Supplement: Supplementary Figure 1 — Multivariate Cox analysis for patients according to the senescence-related subtype stratified by clinicopathological risk factors. (A, B) tumor size, (C, D) tumor grade, (E, F) Tipple-negative breast cancer. [file Image_1.tif]

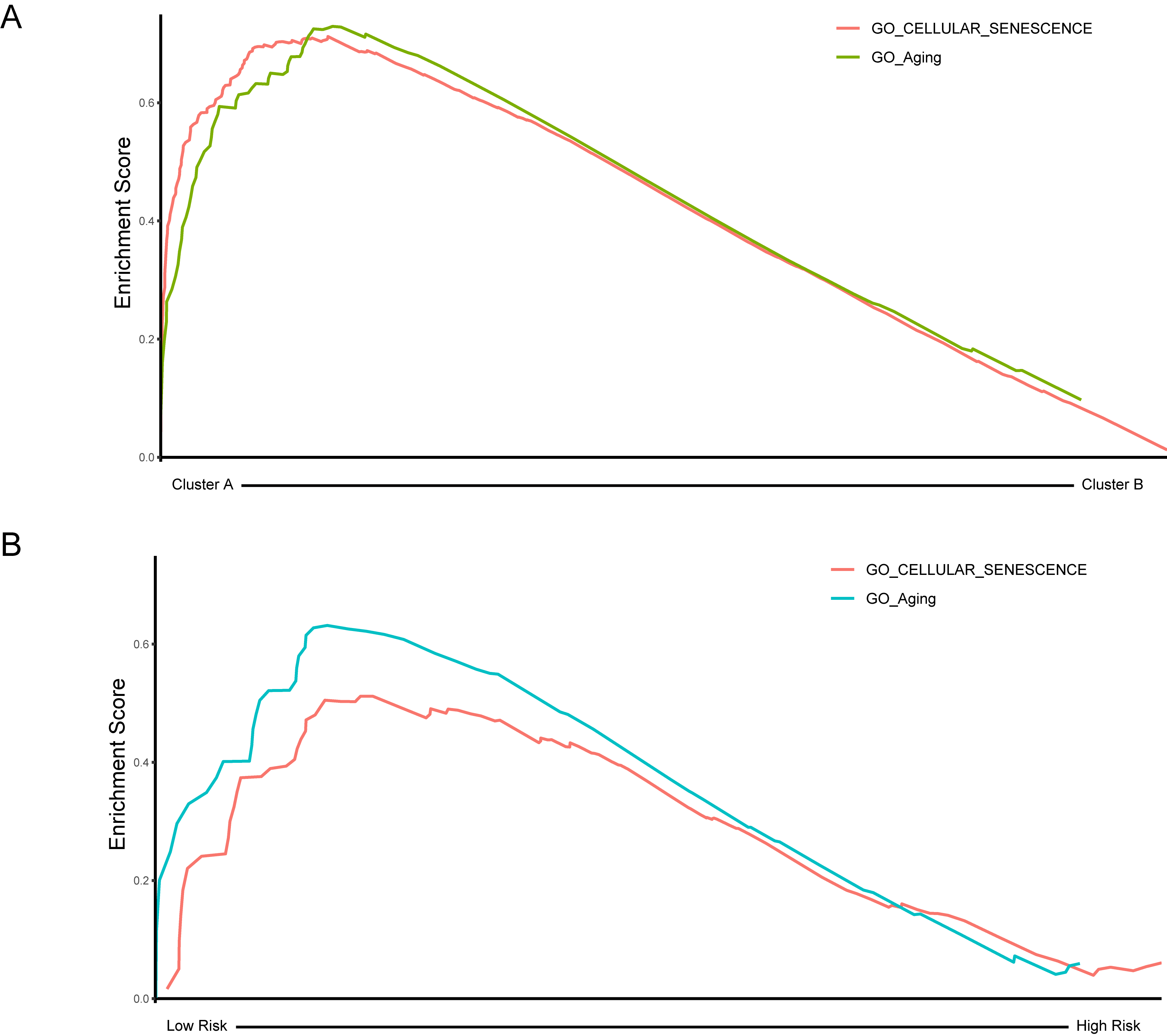

Supplement: Supplementary Figure 2 — Gene set enrichment analysis (GSEA). (A) Cellular senescence and aging were significantly enriched in Cluster A. (B) Cellular senescence and aging were significantly enriched in high risk group. [file Image_2.tif]

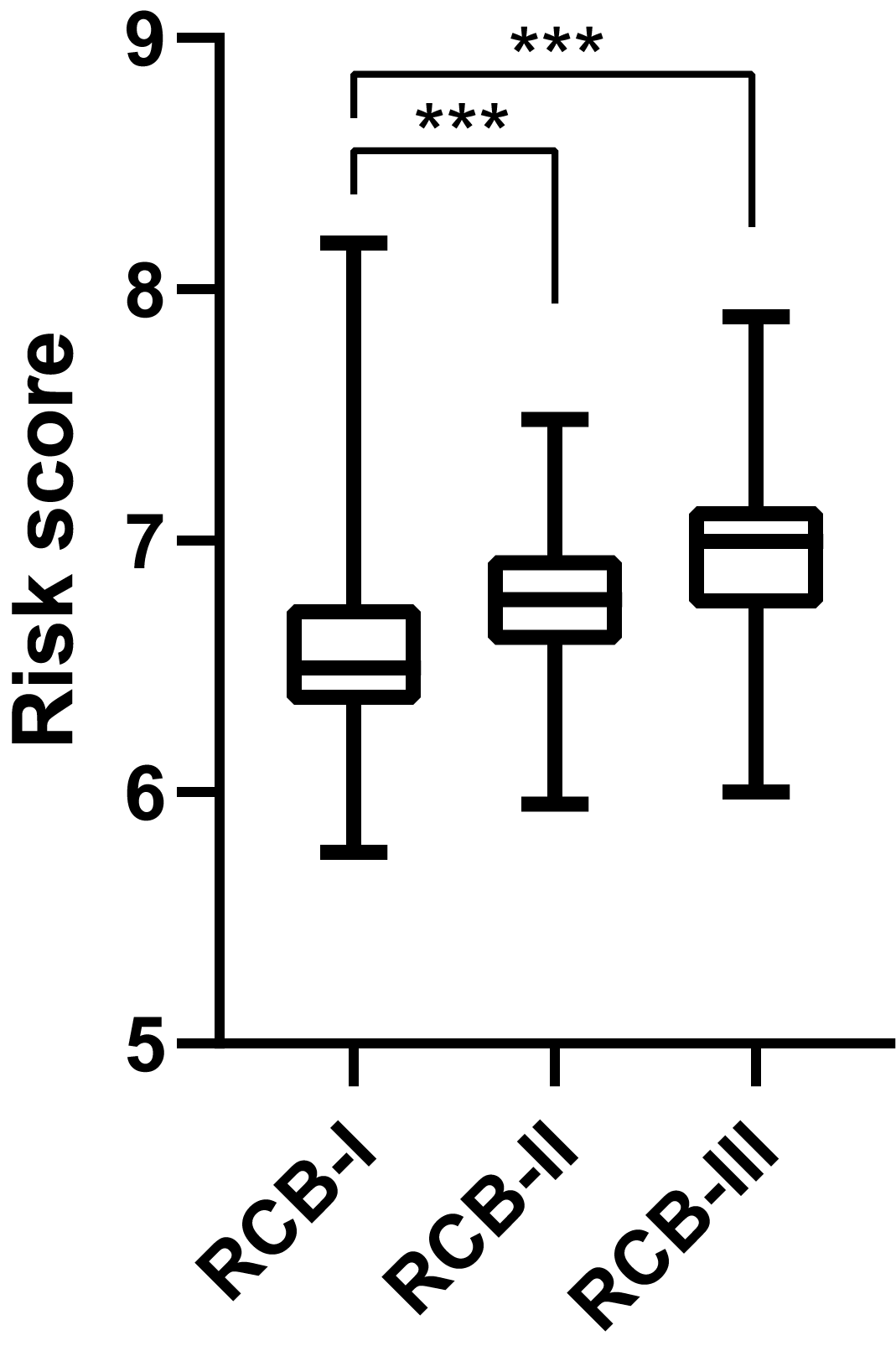

Supplement: Supplementary Figure 3 — Relationships between the senescence-related score and pathological response to neoadjuvant chemotherapy. [file Image_3.tif]
