# Supplementary material for: Identification of senescence-related subtypes, establishment of a prognosis model, and characterization of a tumor microenvironment infiltration in breast cancer
Source: Front Immunol. 2022 Aug 22;13:921182. doi: 10.3389/fimmu.2022.921182 (PMC9441960; doi:10.3389/fimmu.2022.921182)
Supplement: Supplementary file 4 [file DataSheet_1.docx]

| **Table S1. senescence-related genes** | | | | | | | | | |
| --- | --- | --- | --- | --- | --- | --- | --- | --- | --- |
| **AAK1** | **CAVIN1** | **DHCR24** | **HDAC1** | **DDB2** | **KCNJ12** | **MATK** | **P3H1** | **SIRT1** | **TBX2** |
| **ABI3** | **CBX7** | **DHX9** | **HDAC4** | **DEK** | **KDM4A** | **MCL1** | **PAK4** | **SIRT6** | **TERC** |
| **ACLY** | **CBX8** | **DLX2** | **HEPACAM** | **DGCR8** | **KDM5B** | **MCRS1** | **PATZ1** | **SIX1** | **TERF2** |
| **ADCK5** | **CCN1** | **DPY30** | **HIVEP1** | **PROX1** | **KL** | **MDH1** | **PBRM1** | **SLC13A3** | **TERT** |
| **AGT** | **CCND1** | **DUSP16** | **HJURP** | **PRPF19** | **KSR2** | **MECP2** | **PCGF2** | **SLC16A7** | **TFAP4** |
| **AKR1B1** | **CDK1** | **DUSP3** | **HK3** | **PSMB5** | **LATS1** | **MMP9** | **PDCD10** | **SMARCA4** | **TFDP1** |
| **AKT1** | **CDK18** | **E2F1** | **HMGB1** | **PSMD14** | **LEO1** | **MOB3A** | **PDIK1L** | **SMARCB1** | **TGFB1I1** |
| **ALOX15B** | **CDK2AP1** | **EHF** | **HRAS** | **PTTG1** | **LGALS3** | **MORC3** | **PDPK1** | **SMG1** | **TLR3** |
| **AR** | **CDK4** | **ENDOG** | **HSPA5** | **RAD21** | **LIMA1** | **MORF4** | **PDZD2** | **SMURF2** | **TMSB4X** |
| **ARPC1B** | **CDK6** | **EPHA3** | **HSPB2** | **RAF1** | **LIMK1** | **MVK** | **PEBP1** | **SNAI1** | **TNFSF13** |
| **ASF1A** | **CDKN1A** | **ERRFI1** | **ID1** | **RB1** | **MAD2L1** | **MXD4** | **PEX19** | **SOCS1** | **TNFSF15** |
| **ASPH** | **CDKN1B** | **ETS1** | **ID4** | **RBP2** | **MAGEA2** | **MYC** | **PIAS4** | **SOD1** | **TOP1** |
| **ATF7IP** | **CDKN1C** | **ETS2** | **IFNG** | **RBX1** | **MAGOH** | **MYLK** | **PIK3C2A** | **SORBS2** | **TP53** |
| **ATM** | **CDKN2A** | **EWSR1** | **IGFBP1** | **RNASEL** | **MAGOHB** | **NADK** | **PIK3R5** | **SOX2** | **TP63** |
| **AURKA** | **CDKN2AIP** | **EZH2** | **IGFBP3** | **RPS6KA6** | **MAP2K1** | **NANOG** | **PIM1** | **SOX5** | **TPR** |
| **AXL** | **CDKN2B** | **FASTK** | **IGFBP5** | **RSL1D1** | **MAP2K2** | **NDRG1** | **PKM** | **SP1** | **TRIM28** |
| **BAG3** | **CEBPB** | **FBXO31** | **IGFBP6** | **RUNX1** | **MAP2K3** | **NEK1** | **PLA2R1** | **SPIN1** | **TRPM8** |
| **BCL6** | **CENPA** | **FOS** | **IL1A** | **RUVBL2** | **MAP2K6** | **NEK4** | **PML** | **SPOP** | **TXN** |
| **BHLHE40** | **CHEK1** | **FOXM1** | **ING1** | **SELENOH** | **MAP2K7** | **NEK6** | **PMVK** | **SRC** | **TXNIP** |
| **BLK** | **CIP2A** | **FOXO3** | **ING2** | **SENP1** | **MAP3K6** | **NFE2L2** | **PNPT1** | **SREBF1** | **TYK2** |
| **BLVRA** | **CKB** | **FXR1** | **IRF3** | **SENP2** | **MAP3K7** | **NINJ1** | **POT1** | **SRSF1** | **UBTD1** |
| **BMI1** | **CPEB1** | **G6PD** | **IRF5** | **SENP7** | **MAP4K1** | **NOTCH3** | **POU5F1** | **STAT5B** | **USP1** |
| **BRAF** | **CSNK1A1** | **GATA4** | **IRF7** | **SERPINE1** | **MAPK12** | **NOX4** | **PPM1B** | **STK32C** | **VEGFA** |
| **BRCA1** | **CSNK2A1** | **GKN1** | **ITGB4** | **SFN** | **MAPK14** | **NR2E1** | **PPM1D** | **STK40** | **VENTX** |
| **BRD7** | **CTNNAL1** | **GLB1** | **ITPK1** | **SGK1** | **MAPKAPK5** | **NTN4** | **PRKCD** | **SUPT5H** | **WNT16** |
| **BTG3** | **CXCL1** | **GNG11** | **ITPKB** | **SIK1** | **MARCHF5** | **NUAK1** | **PRKCH** | **SYK** | **WNT2** |
| **CAV1** | **CXCL8** | **GRK6** | **ITSN2** | **SIN3B** | **MAST1** | **OTX2** | **PRMT6** | **TACC3** | **WRN** |
| \| **WT1** \| \| --- \| | **WWP1** | **XAF1** | **YAP1** | **YPEL3** | **ZFP36** | **ZMAT3** | **ZNF148** |  |  |

| **Table S2. Clinicopathologic characteristics of breast cancer patients** | | |
| --- | --- | --- |
| **Variables** | **GSE21653** | |
| **Age at diagnosis (years)** |  | |
| **≤ 50** | 87 | |
| **> 50** | 165 | |
| **Tumor size** |  | |
| **T1** | 57 | |
| **T2** | 121 | |
| **T3** | 66 | |
| **Unknown** | 8 | |
| **Lymph node status** |  | |
| **Negative** | 116 | |
| **Positive** | 133 | |
| **Grade** |  | |
| **I** | 43 | |
| **II** | 84 | |
| **III** | 121 | |
| **ER status** |  | |
| **Negative** | 110 | |
| **Positive** | 140 | |
| **PR status** |  | |
| **Negative** | 124 | |
| **Positive** | 126 | |
| **HER2 status** |  | |
| **Negative** | 207 | |
| **Positive** | 26 | |
| **Unknown** | 19 | |
| **P53 status** |  | |
| **Wild Type** | 125 | |
| **Mutant** | 68 | |
| **Unknown** | 59 | |
| **Ki67 status** |  | |
| **Negative** | 58 | |
| **Positive** | 142 | |
| **Unknown** | 52 | |
| **Molecular subtype** |  | |
| **Basal** | 75 | |
| **HER2** | 22 | |
| **Luminal A** | 85 | |
| **Luminal B** | 44 | |
| **Normal** | 26 | |
| **Vital status** |  | |
| **Alive** | 169 | |
| **Dead** | 83 | |
| **Median follow-up (Months)** | 52.92 | |
| *ER: estrogen receptor; PR: progesterone receptor;*  *HER2: human epidermal growth factor receptor 2*  *P53: Tumor Protein P53; Ki67: Proliferation Marker Protein Ki-67* | |  |
